# Remaining Visual Field and Preserved Subjective Visual Functioning Prevent Mental Distress in Patients with Visual Field Defects

**DOI:** 10.3389/fnhum.2013.00584

**Published:** 2013-09-18

**Authors:** Carolin Gall, Doreen Brösel, Bernhard A. Sabel

**Affiliations:** ^1^Institute of Medical Psychology, Medical Faculty, Otto-von-Guericke University of Magdeburg, Magdeburg, Germany

**Keywords:** vision-related quality of life, visual field defects, mental health, psychological distress, National Eye Institute Visual-Functioning Questionnaire

## Abstract

**Background:** Patients with visual field defects after visual pathway lesion may experience reduced vision-related quality of life (vrQoL). It has not been clarified how vrQoL impairments contribute to vision-related mental distress.

**Methods:** One hundred and eight subjects with visual field defects caused by optic neuropathies (age *M* = 57.6; SD = 13.7 years) answered the National Eye Institute Visual-Functioning Questionnaire 39 (NEI-VFQ) for vrQoL and the SF-12 Short Form Health Survey for health-related quality of life. A 10 item composite of NEI-VFQ “visual functioning” and 5 items of “mental-health symptoms due to vision problems” were subjected to Rasch analysis. The test battery comprised static and High Resolution Perimetry (HRP). Regression and path analysis were used to investigate associations between QoL, mental distress, and perimetry results.

**Results:** A higher level of “visual functioning” was associated with monocular impairment and a larger remaining visual field compared to binocular impairment. Subjective “visual functioning” but not visual field parameters predicted “mental-health symptoms due to vision problems” which was the only variable associated with the SF-12 mental component score. The SF-12 physical component score was less strongly associated with “mental-health symptoms due to vision problems.” Here, reaction time in HRP and mean threshold in perimetry were additional significant variables. Path analysis revealed a significant path from remaining visual field via visual functioning on mental health.

**Conclusion:** Subjective consequences of visual impairments in everyday life impact mental health rather than “objective” visual function loss as measured by perimetry. Since a higher extent of vrQoL was related to lower levels of mental distress, the maintenance of vrQoL could reduce and prevent mental distress due to vision problems. Patients with persisting visual field defects may benefit from neuropsychological rehabilitation and supportive therapies.

## Introduction

In optic neuropathy the axons of retinal ganglion cells are degenerated which results in visual field defects (You et al., 2013). These defects lead to impairments in activities of daily living such as reading, driving, or overall orientation (Kerkhoff, 1999). Besides the impaired overall well-being and quality of life (QoL), the risk for falling and trauma as well as social isolation in older patients increases (Timmis and Pardhan, 2012; Sand et al., 2013). In addition, the burden on the health care systems is tremendous since visually impaired patients are often unable to continue their occupation.

Impairments of vision which impact vision-related quality of life (vrQoL) can be measured with the National Eye Institute Visual-Functioning Questionnaire (NEI-VFQ) (Mangione et al., 2001; Franke and Gall, 2008; Labiris et al., 2008). Designed for the assessment of ophthalmologic diseases (Mangione et al., 2001) the NEI-VFQ is a valid and reliable instrument which was frequently used in studies focusing on the impact of visual field defects on subjective vrQoL after central visual pathway lesions (Cole et al., 2000; Beck et al., 2004; Raphael et al., 2006; Papageorgiou et al., 2007; Gall et al., 2009, 2010; Wagenbreth et al., 2011; Galetta et al., 2012; Walter et al., 2012; Jasse et al., 2013). It was demonstrated that diminished vrQoL was significantly related to the size of the visual field defect (e.g., Gall et al., 2009).

Additionally a number of studies have shown that the incidence of neuropsychiatric conditions is high in visually impaired patients with age-related macular degeneration (AMD) (Brody et al., 2001; Mathew et al., 2011), diabetic retinopathy (Cox et al., 1998; Robertson et al., 2006; Hahm et al., 2008; Trento et al., 2013), refractive error (Owsley et al., 2007), myopia (Angi et al., 1996; Rupolo et al., 1997), and amblyopia (Koklanis et al., 2006). The presence of visual field defects is a risk factor for mental distress, especially for depressive symptoms (Zhang et al., 2013). Symptom severity and emotional distress due to vision loss were related to self-reported disability in low vision patients (Dreer et al., 2008).

Mathew et al. (2011) recently found that AMD led to depressive symptoms indirectly via reduced health-related quality of life (hrQoL) ratings obtained with the SF-36 Health Survey. Similarly, in patients with postchiasmatic lesions it was shown that the extent of the visual field defect itself did not directly influence perceived psychological distress, since SCL-90-R symptoms were only reported when diminished vrQoL was present (Gall et al., 2012). The present study aims at extending the previous work by investigating to what extent decreased vrQoL contributes to vision-related mental-health symptoms in patients with optic neuropathies.

## Patients and Methods

### Sample

Data of 108 patients with optic neuropathies who took part in baseline diagnostics of two clinical trials on non-invasive brain stimulation from 2007 to 2011 were analyzed. The studies differed in the way the questionnaires were answered. Some were filled out at home (Sabel et al., 2011a), whereas the majority was answered in an interview at our institute (Gall et al., 2013). Pooling the data of these studies was justified since there were no differences with respect to NEI-VFQ subscales of interest (*p* > 0.40) or SF-12 component scores (*p* > 0.07). Furthermore, the two studies did not differ with respect to lesion age [χ^2^(2, *n* = 108) = 0.36, *p* = 0.84] or sex [χ^2^(1, *n* = 108) = 0.03, *p* = 0.86]. Table 1 gives a description of the sample. The main inclusion criterion was the presence of visual field loss due to optic neuropathies with a lesion age of at least 6 months.

**Table 1:** **Description of patient sample**.

Sample size	108
Age (years), *M* ± SD	57.58 ± 13.67
Male *n* (%)	62 (57.4)
LESION AGE*	
6–12 months *n* (%)	15 (13.9)
1–2 years *n* (%)	11 (10.2)
>2 years *n* (%)	82 (75.9)
**TYPE OF LESION** ***n*** (%)*	
Optic atrophy of unknown etiology	6 (5.6)
Glaucoma	34 (31.5)
AION	24 (22.2)
Optic neuritis	12 (11.2)
Optic nerve compression (tumor)	8 (7.4)
NAION	17 (15.7)
Multiple sclerosis with optic neuritis	1 (0.9)
Optic atrophy after optic disk swelling	3 (2.8)
Optic atrophy after papillitis	1 (0.9)
Optic atrophy after papilledema	1 (0.9)
Leber’s hereditary optic neuropathy	3 (2.8)
Stroke/thrombosis with optic atrophy	5 (4.6)
Congenital optic atrophy	3 (2.8)
**AFFECTED EYES** ***n*** **(%)**	
Monocular impairment, one eye intact	41 (38.0)
Binocular impairment, both eyes defect	58 (53.7)
Binocular impairment, one eye blind	9 (8.3)
**VISUAL PARAMETERS** ***M*** ** ± SD**	
Detection accuracy in HRP (%)	48.6 ± 27.7
Detection accuracy central 5° in HRP (%)	58.1 ± 28.3
Reaction time in HRP (ms)	532 ± 84
Foveal threshold (dB) in static perimetry	21.2 ± 8.9
Mean threshold (dB) in static perimetry	10.9 ± 6.4

^*^ Because of dual diagnoses the sum of lesion age types and etiologies (%) is above 100%.

All patients gave informed consent to participate in above mentioned clinical studies from which the present data were obtained. These clinical studies were previously approved by the local ethics committee and carried out in compliance with ethical standards of the Declaration of Helsinki (1964).

### Diagnosis of visual field defects

Visual field defects were assessed with monocular static standard-automated perimetry using a Twinfield Oculus perimeter (Oculus, Lynnwood, WA, USA) and High Resolution Perimetry (HRP; Kasten et al., 1998) both with a combined chin-forehead-rest.

Monocular static perimetry was performed at a distance of 33 cm. Eye movements were monitored by a camera. Targets (size: III/4 mm^2^, white, luminance: 318 cd/m^2^/0db, duration: 0.2 s, fast threshold strategy, 53 positions) were presented on a background with a constant luminance of 10 cd/m^2^. Patients had to push a button whenever they detected a target.

**Figure 1:**
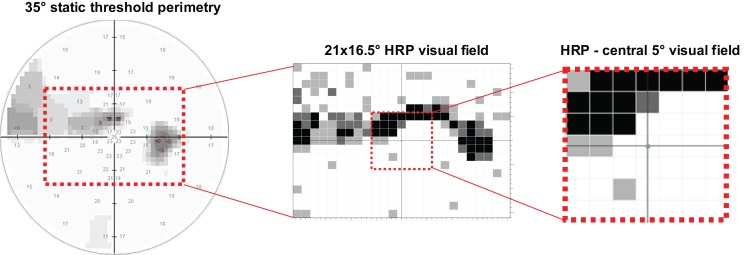
**Visual field measurements were obtained with standard static perimetry and high resolution perimetry (HRP)**. In static perimetry the foveal and the mean threshold (dB) of 53 test positions were analyzed. In HRP the number of correctly detected stimuli was registered in a grid of 25 × 19 test positions as measure of the remaining visual field within a test area of 21° horizontally × 16.5° vertically. By overlapping three HRP tests, visual field areas were categorized as intact (white spots: 3/3 detected stimuli at the respective position), partially (relative) damaged (gray spots: 1/3 or 2/3 detected stimuli), or absolutely impaired (black spots: 0/3 detected stimuli). The 5°-area of HRP was separately analyzed to evaluate the impact of remaining central visual field.

Monocular HRP (25 × 19 grid) was conducted at a distance of 42 cm on a 17^′′^ monitor. Subjects were instructed to press the space bar when a light stimulus or an isoluminant change in the color of the fixation point occurred. The percentage of detected color changes of the fixation point served as reliability parameter. The percentage of correctly detected stimuli was registered as measure of the remaining visual field in the total test area of HRP and in the central 5° (Figure 1). In case detection accuracy was larger than 95% in HRP the visual field was considered as intact. Additionally, mean reaction time (ms) was recorded.

### QoL instruments

The validated German 39-item version of the NEI-VFQ (Franke et al., 1998) was administered. The instrument is well suited for patients with central visual pathway lesions (see Introduction). The questionnaire consists of 39 rating items with 12 subscales which were recently subjected to missing validity (Dougherty and Bullimore, 2010; Marella et al., 2010). As suggested by Rasch models a “visual functioning” scale consisting of 10 items according to Marella et al. (2010) was calculated for vrQoL. For the purpose of the present study the items of the original subscale “mental-health symptoms due to vision problems” were combined to a second scale of interest: (1) How much of the time do you worry about your vision? (2) I feel frustrated a lot of the time because of my vision, (3) I have much less control over what I do, because of my vision, (4) I worry about doing things that will embarrass myself or others, because of my vision, and (5) I am often irritable because of my vision. Rasch analysis was applied to both scales.

The German Short Form Health Survey SF-36 or SF-12 was used to obtain measures of hrQoL (Bullinger and Kirchberger, 1998). The SF-12 was selected because it is more economical while maintaining a high correlation to the original SF-36 scores (Gandek et al., 1998). In contrast to the NEI-VFQ, validation studies of the German SF-36 revealed similar intervals between response categories (Bullinger and Kirchberger, 1998; Forero et al., 2013). Therefore the items were treated as interval and combined to the physical component score (PCS) and mental component score (MCS) according to the manual.

### Statistical analysis

Demographic and clinical data were analyzed with SPSS 21.0 for Windows (IBM Deutschland GmbH, Ehningen). In order to ensure interval-scaled NEI-VFQ item responses for subsequent analyses WINMIRA 2001 (Scienceplus, Groningen) was used to calculate an ordinale Rasch analysis for the two NEI-VFQ scores of “visual functioning” (*n* = 103) and “mental-health symptoms due to vision problems” (*n* = 108). Response categories were inverted from zero to four in a way that a higher response category represents a higher ability, respectively a better QoL. The equidistance model without distribution smoothing was applied because equal response distances for items independent of their response dimension (agreement, frequency, or intensity) were not expected. For evaluation of model fit Pearson χ^2^ was calculated. Usefulness of individual items and response categories were rated according to category thresholds and *Q* indices for discrimination. Person abilities were estimated using warms weighted likelihood estimate (WLE). Because of the small sample size the number of different response patterns is presumably small. Therefore, the analysis should be restricted to this study and χ^2^ is interpreted carefully. Furthermore bootstrapping (400 samples) was chosen to evaluate the fit of the Rasch model for the simulated distribution of the calculated parameters.

A first multiple linear regression with backward inclusion: (i) was applied to analyze the effects of different visual field parameters on NEI-VFQ “visual functioning.” The following visual field parameters were considered relevant: detection accuracy in HRP (%), detection accuracy within central 5° in HRP (%), reaction time in HRP (ms), foveal threshold (dB) in static perimetry, mean threshold (dB) in static perimetry, and ocular functional state. The ocular functional state was included since in patients with monocular visual impairment the intact eye may compensate the resulting vision loss. The presence of one intact eye was used as reference for dummy coding of the ocular functional state (monocular vision impairment with one intact eye, binocular vision impairment with residual vision on both eyes, and binocular vision impairment with one blind eye).

Three further multiple regression were calculated to identify potential predictors of: (ii) “mental-health symptoms due to vision problems” (NEI-VFQ), (iii) mental aspects of hrQoL (MCS of the SF-12), and (iv) physical aspects of hrQoL (PCS of the SF-12). Predictors were above mentioned visual field parameters, NEI-VFQ “visual functioning,” and for regression (iii) and (iv) the NEI-VFQ “mental-health symptoms due to vision problems” scale.

A pairwise exclusion of missing data was selected for all regressions.

For path analysis [SPSS Amos 21 (IBM Deutschland GmbH, Ehningen)] a maximum likelihood estimation was calculated to examine direct and indirect effects of HRP detection accuracy on “mental-health symptoms due to vision problems” via vrQoL. Missing data were estimated and error regression weights were fixed to one. χ^2^, CFI, and RMSEA were used as fit indices. *p*-Values < 0.05 were considered as statistically significant.

## Results

**Figure 2:**
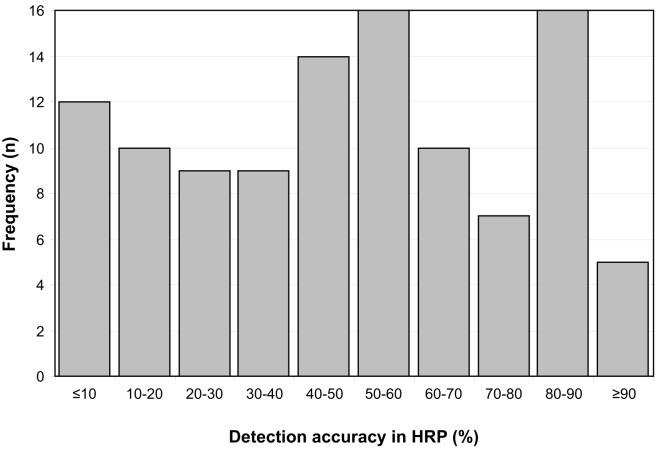
**Frequency distribution of detection accuracy in HRP as a measure of remaining visual field**. Detection accuracy values below 30% reflect severe damage, 30–70% moderate, and more than 70% mild visual field damage.

Detection accuracy in HRP as a measure of remaining visual field varies between 0.14 and 94.02%. The frequency distribution of measured detection accuracy is depicted in Figure 2.

The mean NEI-VFQ “visual functioning” score was 71.45 (SD = 20.86). Patients reported a mean score of “mental-health symptoms due to vision problems” of 66.20 (SD = 21.55) which was lowered in a clinically relevant extent when compared to healthy controls (Labiris et al., 2008; Wagenbreth et al., 2011).

Neither sex nor lesion age significantly influenced “mental health due to vision problems” (sex, *U* = 1328, *p* = 0.54; lesion age, Kruskal–Wallis χ^2^(2) = 2.96, *p* = 0.23). Most pronounced subjective impairments were reported for item 3 “worries about vision” and item 25 “worry to embarrass myself/others.” The remaining items had similar response distributions with approximately two thirds of patients reporting no significant impairment.

The Rasch model fitted for the NEI-VFQ “visual functioning” and “mental-health symptoms due to vision problems” scale (*p* ≥ 0.24). Bootstrapping revealed no significant deviation from Rasch model (*p* ≥ 0.52). The mean item location of NEI-VFQ “visual functioning” was 0.00 (SD = 0.86) and mean person location was 1.76 (SD = 1.85). Item locations varied from the easiest to the most difficult item between −1.76 (“picking out and matching own clothes”) and 0.96 (“reading ordinary print in newspapers”). All thresholds were ordered without overlaps. According to the *Q* index there was no significant deviation in discrimination compared to the Rasch model (*p* > 0.19). The averaged reliability coefficient was 0.92.

**Figure 3:**
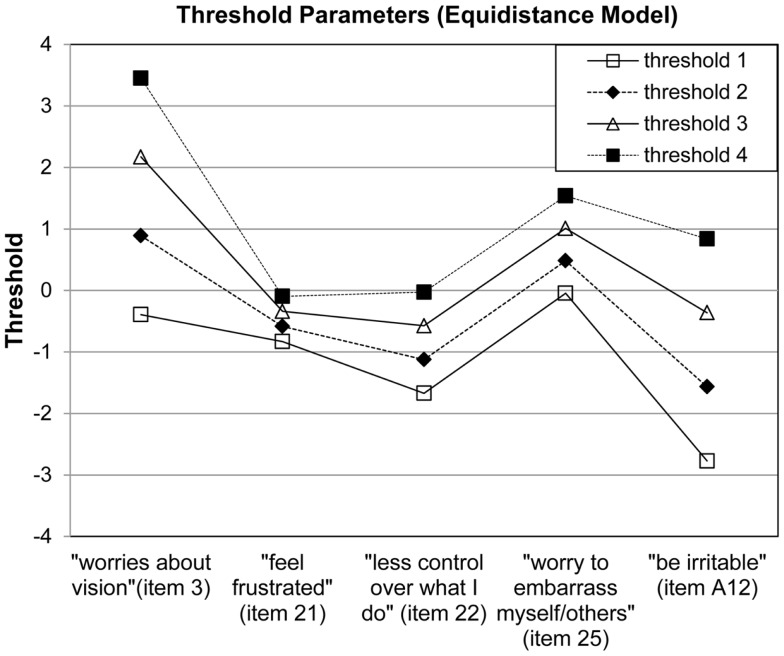
**Visual inspection of threshold parameters reveal ordered thresholds for each NEI-VFQ item assessing “mental-health symptoms due to vision problems”**. For Rasch analysis items were inverted in a way that a higher response category represents a higher ability.

**Figure 4:**
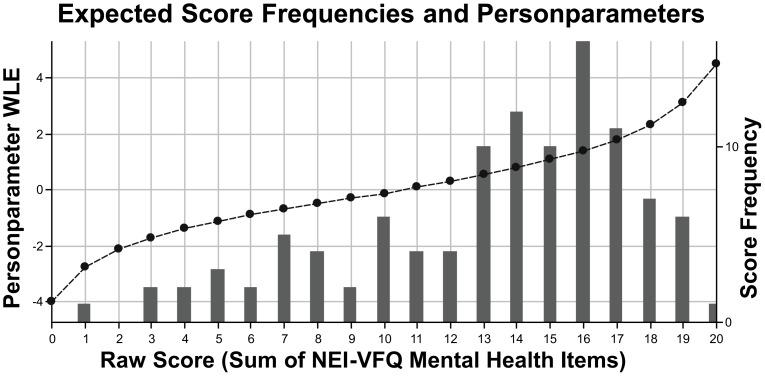
**Expected response frequencies and person parameters (weighted likelihood estimation, WLE) for self-reported “mental-health symptoms due to vision problems” assessed with the respective NEI-VFQ subscale (sum score of five items)**. Person parameters (dotted line) lay between −4.00 and 4.49.

The subscale “mental-health symptoms due to vision problems” showed a good fit to the model (χ^2^ = 3150.44, *p* = 0.24) with a mean item location of 0.00 (SD = 1.09) and mean person location of 0.38 (SD = 0.23). Item locations reached from −0.96 (“being irritable”) to 1.53 (“worries about vision”). All item thresholds were ordered without any overlap (Figure 3). No significant deviations in discrimination could be observed (*p* > 0.46). Response frequencies for NEI-VFQ items assessing “mental-health symptoms due to vision problems” and person parameters are illustrated in Figure 4. Reliability was 0.79.

Regression results of visual field parameters on NEI-VFQ “visual functioning” are shown in Table 2. The only significant predictors in the regression of NEI-VFQ “visual functioning” on visual field parameters were the ocular functional state and HRP detection accuracy with an adjusted *R*^2^ of 0.37. The model implies that patients with binocular impairment but some preserved vision on both eyes reported a subscale score for “visual functioning” which was about 14.23 units lower than in the case of monocular visual impairment. Patients with one blind eye reported a 29.24 units lower score. An increase of 1 unit in HRP detection accuracy was accompanied by an increase in NEI-VFQ “visual functioning” score of 0.29 units.

**Table 2:** **Summary of results from backward multiple linear regression models**.

	NEI-VFQ “visual functioning”		NEI-VFQ “mental-health symptoms due to vision problems”		SF-12 MCS		SF-12 PCS	
	[*R*_adj_^2^ = 0.37, *F*(3,94) = 20.08, *p* < 0.01]		[*R*_adj_^2^ = 0.38, *F*(1,96) = 60.75, *p* < 0.01]		[*R*_adj_^2^ = 0.30, *F*(1,93) = 41.34, *p* < 0.01]		[*R*_adj_^2^ = 0.18, *F*(4,90) = 6.31, *p* = 0.01]	
	ß	*p*	ß	*p*	ß	*p*	ß	*p*
Detection accuracy in HRP	*0.38*	<*0.01*	0.01	0.88	−0.11	0.24	0.16	0.47
Detection accuracy central 5° in HRP	0.15	0.39	−0.01	0.92	−0.12	0.19	−0.04	0.78
Reaction time in HRP	0.04	0.78	0.02	0.84	0.03	0.77	−*0.38*	*0.01*
Foveal threshold in static perimetry	0.11	0.26	0.05	0.53	−0.01	0.93	−0.10	0.41
Mean threshold in static perimetry	0.02	0.91	0.07	0.41	−0.14	0.12	−*0.43*	<0.01
Binocular impairment, both eyes residual vision	*−0.34*	<*0.01*	−0.11	0.19	0.09	0.34	0.01	0.90
Binocular impairment, one blind eye	*−0.39*	<*0.01*	0.07	0.41	−0.01	0.90	−0.17	0.08
NEI-VFQ visual functioning			*0.62*	<*0.01*	0.04	0.71	0.07	0.96
NEI-VFQ mental-health problems					*0.55*	<*0.01*	*0.37*	<*0.01*

p  < 0.05 in italics. Intercorrelations between visual field parameters in the regression for PCS lead to a negative ß-value for the mean threshold in static perimetry.

The regression of visual field parameters and NEI-VFQ “visual functioning” on “mental-health symptoms due to vision problems” yielded only “visual functioning” as significant predictor with an adjusted *R*^2^ of 0.38 (Table 2). An increase of 1 unit in the “visual functioning” score was related to an increase of 0.64 units in the “mental-health symptoms due to vision problems” score.

Regression of visual field parameters and NEI-VFQ results on MCS and PCS was calculated separately. For hrQoL mean SF-12 MCS was 54.12 (SD = 7.98) and mean PCS 48.22 (SD = 8.02) and thus significantly lowered with score differences of more than 25 compared to healthy control values from Bullinger and Kirchberger (1998). The NEI-VFQ score of “mental-health symptoms due to vision problems” was significantly associated with the MCS with an adjusted *R*^2^ of 0.30 (Table 2). An increase of 1 unit in “mental-health symptoms due to vision problems” led to an increase of 0.21 units in MCS. The model of the PCS demonstrated similar results but a less distinct association with “mental-health symptoms due to vision problems” with an adjusted *R*^2^ of 0.10. An increased score of “mental-health symptoms due to vision problems” by one led to an increase of 0.14 units in PCS. For the PCS reaction time in HRP and mean threshold in static perimetry were significant predictors as well. The adjusted *R*^2^ for all significant predictors was 0.18 (Table 2). A rise of 1 unit in reaction time led to a decrease of 0.04 units in PCS. Similarly, an improvement about 1 unit in mean threshold in static perimetry was accompanied by a decrease of 0.54 units in PCS. Since some parameters were highly intercorrelated, the regression weight of the mean threshold was negative.

**Figure 5:**
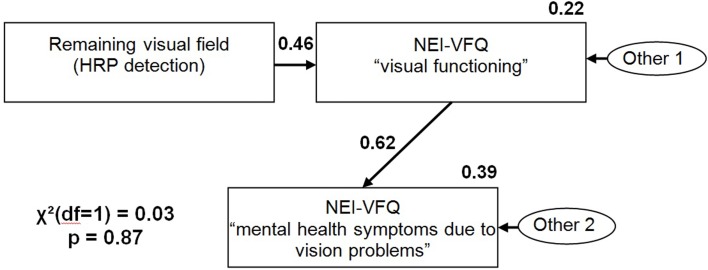
**Path model with HRP detection accuracy and NEI-VFQ “visual functioning” as exogenous variables and NEI-VFQ “visual functioning” and NEI-VFQ “mental-health symptoms due to vision problems” as endogenous variables**.

Path analysis revealed that HRP detection accuracy as a measure of remaining visual field, predicts “visual functioning,” i.e., vrQoL (*R*^2^ = 0.22, ß = 0.46, *p* < 0.01) which in term is related to “mental-health symptoms due to vision problems” (*R*^2^ = 0.39, ß = 0.62, *p* < 0.01). There is no significant path between HRP detection accuracy and “mental-health symptoms due to vision problems” (Figure 5). The model had a good data fit [χ^2^(1, *n* = 108) = 0.03, *p* = 0.87, CFI = 1.00, RMSEA = 0.00].

## Discussion

With the present study we wished to determine to what extent vrQoL decrements contribute to vision-related mental-health symptoms. There were clinically relevant reductions of roughly 20 points concerning the NEI-VFQ subscale “mental-health symptoms due to vision problems” when comparing patients with optic neuropathies to recently published healthy control samples without visual impairments (Labiris et al., 2008; Wagenbreth et al., 2011). The most serious restrictions of mental health were observed when asking for worries about vision which is in agreement with a previous study showing that NEI-VFQ was associated with phobic anxiety in the SCL-90-R (*r* = −0.64, *p* < 0.01; Gall et al., 2012). In the present sample, the NEI-VFQ “visual functioning” score was severely reduced as well.

Only the ocular functional state and HRP detection were related to the “visual functioning” score. It may be concluded that monocular blindness is associated with more severe restrictions in everyday activities that require some preserved binocular vision. As expected a higher degree of remaining visual field was associated with a better score in subjective “visual functioning” and therefore with fewer restrictions in everyday life. Indeed visual field parameters were not found to be appropriate predictors for mental-health concerns. Only NEI-VFQ “visual functioning” as a measure of vrQoL significantly predicted “mental-health symptoms due to vision problems.” This finding could be expected since both subscales are part of the same questionnaire.

As hypothesized, the proportion of explained variance by “mental-health symptoms due to vision problems” was higher for mental aspects of hrQoL (MCS of SF-12: 30%) than for physical aspects of hrQoL (PCS of SF-12: 10%) with higher ß-values for MCS. For PCS reaction time in HRP and mean threshold in static perimetry were significant predictors as well resulting in an increase from 10 to 18% of explained variance. Here, further parameters related to physical comorbidities are likely to have an influence especially when an elderly sample is examined.

Using path analysis an indirect effect of remaining visual field on mental-health problems via vrQoL was shown. This supports the assumption that subjective consequences of visual impairments in everyday life impact mental health rather than “objective” visual function loss as measured in clinical settings. Similarly, in a study by Zhang et al. (2013) subjective perception of visual field impairments correlated with depression whereas visual acuity was uncorrelated. We have recently shown that mental distress (measured with the SCL-90-R) was related to diminished vrQoL, but it was actually not influenced by the extent of the visual field loss itself (Gall et al., 2012) which is in agreement with the results of the path analysis in the present study. Since regression and path analysis are not suitable to draw conclusions about causality, a model with inverse directions would fit, too. This also implies that vision-impaired patients who are at risk for mental-health symptoms more likely suffer from reduced vrQoL due to the visual field defect.

The obtained results are limited in several ways. Firstly, we have studied a self-selected sample that participated in a clinical trial with certain inclusion criteria. Furthermore, Rasch analysis was conducted to ensure interval-scaled NEI-VFQ item responses for subsequent analyses. The NEI-VFQ scales subjective “visual functioning” suggested by Marella et al. (2010) and “mental-health symptoms due to vision problems” both fitted with Rasch. However, we have studied only a small sample and observed only a restricted number of different response patterns. Bootstrapping allowed a more accurate decision about the model fit but a larger sample size should be used to minimize this problem. Even when the data fit to a proposed model, as in our study, it is not necessarily the best model. Regression enabled to predict only 18–38% of observed variance. VrQoL is a multidimensional construct by definition. To be able to obtain a deeper understanding of mental-health symptoms in vision loss the number and content of items capturing mental-health symptoms should be expanded to more precisely evaluate psychological distress that is associated with vision loss.

Further analyses should consider more complex models to avoid an omitted variable bias (Menard, 2002). Instead of using averaged results of monocular visual field tests, binocular visual fields are possibly more strongly related to subjective vision. Also temporal processing deficits in the intact visual field may turn out to be a relevant variable (Bola et al., 2013). Psychological variables, like individual coping strategies or social resources, could be considered as possible confounders (Boerner and Wang, 2012). The finding that elderly subjects with vision impairments had a poorer level of depression, anxiety, and hrQoL despite the highest level of social support (Kempen et al., 2012) points to the importance of including measures of social support in the model.

There is evidence that diminished vrQoL is significantly related to the severity of the visual field loss though the correlation is weak to moderate (Papageorgiou et al., 2007; Gall et al., 2009). Reducing visual field loss by means of vision restoration techniques is an important prerequisite to preserve or improve vrQoL. This again may constitute a protective factor in maintaining mental well-being despite the visual impairment. Participation in visual rehabilitation and its early onset appears to have a preventive effect on mental-health problems such as secondary depression in AMD (Mielke et al., 2013). Since the minimum lesion age in these studies was 6 months and none of our patients received visual rehabilitation prior to data acquisition we could not investigate the potential effect of (early) rehabilitation onset on preservation of vrQoL which is also an issue for future research. In fact, neuropsychological training to reduce the visual field defect (Vision Restoration Training, e.g., Sabel et al., 2011b) or to improve visual scanning behavior in the area of partial blindness (eye movement training, e.g., Nelles et al., 2010) are well known. It has not been studied yet if patients with optic neuropathies develop compensatory strategies on their own or receive other advice from low vision services or eye specialists that may transfer into effective coping strategies in everyday life and support to maintain vrQoL and mental health.

To improve health care for patients with optic neuropathies, visual functional improvements should not be the only aim but also the maintenance of vrQoL together with an amelioration of related mental-health symptoms. Proper assessment of the respective symptoms together with systematic QoL assessment is needed to identify the psychological needs of these patients. Additionally the offer of psychosocial treatments should be regularly provided for patients at risk for low QoL and high levels of mental distress. The treatment of the visually impaired should be an integrative approach encompassing vision restoration and/or compensation treatments, teaching of effective coping strategies and, if necessary, supportive psychotherapy aimed at reducing anxiety and depression. When vision restoration is not sufficiently achieved, the aim should be to enhance vrQoL to reduce mental distress even if vision loss cannot be further improved.

## Conflict of Interest Statement

The authors declare that the research was conducted in the absence of any commercial or financial relationships that could be construed as a potential conflict of interest.
